# Taxes and Subsidies for Improving Diet and Population Health in Australia: A Cost-Effectiveness Modelling Study

**DOI:** 10.1371/journal.pmed.1002232

**Published:** 2017-02-14

**Authors:** Linda J. Cobiac, King Tam, Lennert Veerman, Tony Blakely

**Affiliations:** 1 Centre for Health Policy, School of Population and Global Health, University of Melbourne, Melbourne, Victoria, Australia; 2 School of Public Health, The University of Queensland, Herston, Queensland, Australia; 3 Burden of Disease Epidemiology, Equity and Cost-Effectiveness Programme, Department of Public Health, University of Otago, Wellington, Wellington, New Zealand; Stanford University, UNITED STATES

## Abstract

**Background:**

An increasing number of countries are implementing taxes on unhealthy foods and drinks to address the growing burden of dietary-related disease, but the cost-effectiveness of combining taxes on unhealthy foods and subsidies on healthy foods is not well understood.

**Methods and Findings:**

Using a population model of dietary-related diseases and health care costs and food price elasticities, we simulated the effect of taxes on saturated fat, salt, sugar, and sugar-sweetened beverages and a subsidy on fruits and vegetables, over the lifetime of the Australian population. The sizes of the taxes and subsidy were set such that, when combined as a package, there would be a negligible effect on average weekly expenditure on food (<1% change). We evaluated the cost-effectiveness of the interventions individually, then determined the optimal combination based on maximising net monetary benefit at a threshold of AU$50,000 per disability-adjusted life year (DALY). The simulations suggested that the combination of taxes and subsidy might avert as many as 470,000 DALYs (95% uncertainty interval [UI]: 420,000 to 510,000) in the Australian population of 22 million, with a net cost-saving of AU$3.4 billion (95% UI: AU$2.4 billion to AU$4.6 billion; US$2.3 billion) to the health sector. Of the taxes evaluated, the sugar tax produced the biggest estimates of health gain (270,000 [95% UI: 250,000 to 290,000] DALYs averted), followed by the salt tax (130,000 [95% UI: 120,000 to 140,000] DALYs), the saturated fat tax (97,000 [95% UI: 77,000 to 120,000] DALYs), and the sugar-sweetened beverage tax (12,000 [95% UI: 2,100 to 21,000] DALYs). The fruit and vegetable subsidy (−13,000 [95% UI: −44,000 to 18,000] DALYs) was a cost-effective addition to the package of taxes. However, it did not necessarily lead to a net health benefit for the population when modelled as an intervention on its own, because of the possible adverse cross-price elasticity effects on consumption of other foods (e.g., foods high in saturated fat and salt). The study suggests that taxes and subsidies on foods and beverages can potentially be combined to achieve substantial improvements in population health and cost-savings to the health sector. However, the magnitude of health benefits is sensitive to measures of price elasticity, and further work is needed to incorporate potential benefits or harms associated with changes in other foods and nutrients that are not currently modelled, such as red and processed meats and fibre.

**Conclusions:**

With potentially large health benefits for the Australian population and large benefits in reducing health sector spending on the treatment of non-communicable diseases, the formulation of a tax and subsidy package should be given a more prominent role in Australia’s public health nutrition strategy.

## Abstract 2

Linda Cobiac and colleagues present a cost effectiveness modelling study where a sugar tax combined with subsidies for fruit and vegetables would improve health for the Australian population and reduce health sector spending.

## Author Summary

**Why Was This Study Done?:**

- A growing number of countries have implemented or are considering implementing food and drink taxes or subsidies to address the growing burden of dietary-related diseases.
- However, the long-term population health impact and cost-effectiveness of combining unhealthy food taxes and healthy food subsidies are not well understood.

**What Did the Researchers Do and Find?:**

- We modelled the potential impact for the Australian population of five policy options: taxes on saturated fat, salt, sugar, and sugar-sweetened beverages and a subsidy on fruits and vegetables.
- The simulations indicated that the combination of the taxes and subsidy could avert as many as 470,000 disability-adjusted life years (95% uncertainty interval: 420,000 to 510,000) in the Australian population of 22 million, at a net cost-saving of AU$3.4 billion (AU$2.4 billion to AU$4.6 billion; US$2.3 billion) to the health sector.
- The sugar tax produced the biggest gains in health, followed by the salt tax, the saturated fat tax, and the sugar-sweetened beverage tax; the fruit and vegetable subsidy, while cost-effective when added to the package of taxes, did not lead to a net health benefit for the population when modelled as an intervention on its own, due to an associated increase in the consumption of other less healthy foods.

**What Do These Findings Mean?:**

- The modelling illustrates the potentially large benefits of combining food taxes and subsidies for improving population health and reducing health sector spending.
- The Australian government should follow the lead of France, Mexico, the UK, and other countries in developing a policy for taxing and subsiding foods and drinks to improve public health.
- While the modelling strongly indicated that there could be benefits with taxing unhealthy products, the health benefits of focusing policy only on subsidising healthy food were more sensitive to modelled data, and we recommend further research to measure and model how populations respond to healthy food subsidies.

## Introduction

The relationship between non-communicable diseases and consumption of unhealthy foods and drinks is well known, with dietary factors contributing almost 10% of the global disease burden [1]. Price is a key driver of food purchasing [2], and experimental studies in real-world environments (e.g., canteens and vending machines) and virtual supermarket-type environments show that people reduce consumption of unhealthy foods when the price of these products is increased [3].

Countries have been experimenting with taxing foods and drinks since the early 1980s [4]. In most countries, taxes have been applied to food and drink items that are clearly unhealthy or a luxury item, such as sugar-sweetened beverages and confectionery, with taxes often applied as a sales tax (e.g., United States) or an import duty (e.g., Fiji, Nauru, and French Polynesia). More recently, countries such as Denmark and Hungary have implemented taxes on a wider range of foods and drinks, including products such as meat and dairy, with taxes based on the levels of saturated fat, sugar, or salt. Mexico also introduced an 8% tax on nonessential high energy density foods and a 10% tax on sugar-sweetened beverages in 2014. Evaluation of the Mexico taxes found a 5% decrease in purchases of taxed foods in the first year, compared with no change in the purchase of untaxed products [5].

Using observations of fluctuations in product prices and changes in household expenditure on foods and drinks, economists have estimated the consumer price elasticity (the change in consumption with change in price) for a wide range of foods and drinks [6–9]. Cost-effectiveness models of taxation interventions using price elasticity data suggest that a 10% tax on unhealthy foods in Australia [10], a 20% tax on sugar-sweetened beverages in Australia [11], and a tax on salt in the US [12] are all potentially cost-saving, leading to large improvements in population health and savings in disease treatment costs.

However, the scope of the health outcome modelling in the previous cost-effectiveness studies was restricted to single dietary outcomes; the Australian studies only modelled the relationship between energy intake and body mass index (BMI), and the US study only modelled the relationship between salt and blood pressure. While cost-effectiveness models have typically been narrowly focused, health models that take a broader range of dietary and health impacts into account suggest that the balance of benefit and harm for food taxes does not always lead to favourable disease outcomes [13]. While most health outcome studies indicate that taxing sugar-sweetened beverages or foods and drinks high in sugar, fat, or salt is effective in reducing energy intake and consumption of the targeted nutrients [2,13], several high-quality studies have shown that a tax or subsidy may have an unfavourable effect on nutrients that are not the intended target (e.g., a decrease in fibre [9]), and this may lead to a net increase rather than decrease in deaths [14,15].

It is possible that taxes or subsidies could be combined in a way that would achieve a net improvement in health and a net reduction in the costs of disease treatment, for example, by combining a saturated fat tax with a subsidy on fruits and vegetables. But methods for deriving an optimal package of taxes and subsidies that takes long-term population health and cost implications into account are yet to be developed. In this study, we use an Australian cost-effectiveness model to evaluate a range of food and drink taxes and subsidies, implemented individually and in all combinations, to determine an optimally cost-effective package of tax and subsidy options.

## Methods

### Tax and Subsidy Scenarios

In this study, we evaluate combinations of five interventions: (1) taxing saturated fat, (2) taxing excess salt in processed foods, (3) taxing sugar-sweetened beverages, (4) subsidising fruits and vegetables, and (5) taxing processed foods high in sugar. We initially set the taxes on sugar-sweetened beverages and saturated fat to be equivalent to the taxes implemented in Denmark [16,17]. Finding no precedent for a tax on salt or subsidy on fruits and vegetables, we set the tax on excess salt (i.e., sodium in excess of maximum recommended levels for food items [18]) and the subsidy on fruits and vegetables to be equivalent in size to the Danish taxes, with adjustment of units to reflect the different food types (e.g., milligrams of sodium instead of kilograms of fat, kilograms of fruits and vegetables instead of litres of soft drink). We then scaled the magnitude of all taxes so that the combination of all five taxes/subsidies had a negligible effect on current average weekly expenditure on food (defined as a change of <1%) and checked that the expenditure effect of any one tax or subsidy also did not exceed 1%. Table 1 gives a description of the five taxation/subsidy scenarios, and S1 Table provides an illustration of the change in price for some typical purchases.

**Table 1:** The food tax and subsidy interventions.

Intervention	Tax or Subsidy	Sources and Assumptions
Saturated fat tax	$1.37/100 g of saturated fat	Tax on saturated fat content in foods with >2.3% saturated fat, excluding drinking milk
Excess salt tax	$0.30/1 g of sodium	Tax on sodium in excess of Australian maximum recommended levels, excluding fresh fruits, vegetables, meats, and dairy products
Sugar-sweetened beverage tax	$0.47/l	Tax on sugar-sweetened soft drinks, energy drinks, cordials, and fruit drinks*
Fruit and vegetable subsidy	$0.14/100 g	Subsidy on all fresh and preserved fruits and vegetables
Sugar tax	$0.94/100 ml of ice cream; $0.85/100 g of sugar	Tax on ice cream containing >10 g of sugar per 100 g of ice cream; tax on sugar content in excess of 10 g per 100 g of all other products, excluding fresh fruits, vegetables, and unflavoured dairy products

All tax and subsidy amounts are shown in 2010 Australian dollars.

*“Fruit drinks” are currently defined in Australia as fruit juice beverages having <90% by volume of fruit juice.

### Change in Product Prices

We estimated the baseline prices of all food and drink products from two surveys of the prices of all product varieties available online from Woolworths Supermarket in June 2011 (winter) and February 2012 (summer). We took the median price from all brands available within each product sub-category (wholemeal bread, white bread, etc.), adjusting prices to 2010 values (baseline year for cost-effectiveness analyses) using the consumer price index for each food [19], and taking the average of the two seasonal surveys.

Using nutrient levels (e.g., sodium, saturated fat) estimated for Australian food and drink products [20], we then determined the change in price of all products with each tax and subsidy scenario.

### Change in Dietary Patterns

From Australian Health Survey data on the consumption of foods and drinks [21], and taking product wastage [22,23] into account, we determined the change in daily intake of foods (e.g., servings of fruits and vegetables) and nutrients (e.g., sodium, saturated fat) for each tax and subsidy scenario, using price elasticity data. In economic analyses, an own-price elasticity measures the change in purchase of a product with a 1% increase in its price, while a cross-price elasticity measures the change in product purchase with a 1% increase in the price of another product.

Price elasticities have been measured in a number of countries, including the UK [24], Denmark [9], US [6], New Zealand [7], and Australia [25]. The authors of the Australian elasticity study had concerns with inaccuracies in their price data; further, their study did not include beverages and was limited to a small number of food categories that are poorly matched with nutritional analyses (all products from grains were categorised into bread or rice, rather than distinguishing between breads and cereals, cakes and biscuits, pastry products, etc.). Given the limitations of the Australian data, we chose to use more recent and comprehensive food price elasticities from New Zealand in our primary analyses, even though we were modelling the health implications of food taxes and subsidies in the Australian population. While price elasticities can be influenced by cultural food preferences and levels of wealth [13], Australia and New Zealand have a common history of British colonisation and later migration, which has led to similar dietary patterns and economic systems. In addition, the close proximity of the two island nations, and their relative isolation from other Western countries, has led to a common range of food products and manufacturers. However, we also repeated the analyses with UK elasticities to explore the sensitivity of the modelling to the choice of elasticity values.

### Change in Risk Factor Exposure

From the modelled shifts in dietary patterns, we determined changes in three disease risk factors: fruit and vegetable intake, systolic blood pressure, and BMI. Baseline prevalence of the risk factors was derived by age and sex from the Australian Health Survey 2011–2012. Change in daily fruit and vegetable intake due to each tax/subsidy intervention was determined directly from the change in dietary intake. Change in systolic blood pressure was determined from the net change in sodium intake across the diet, using regression models derived by Law et al. [26]. While there has been much recent debate about a possible J- or U-shaped relationship between sodium intake and health outcomes, we did not think the evidence sufficiently strong to model an increased risk at lower levels of sodium intake [27,28]. Change in BMI was determined from the modelled change in energy intake, using the formula described by Christiansen and Garby [29], assuming constant height and physical activity, which we estimated, by age and sex, from the Australian Health Survey 2011–2012.

### Change in Disease Incidence

We modelled the change in disease-specific incidence by using the population impact fraction equation [30] to quantify the disease risk reduction associated with each change in risk factor exposure. We modelled the effects of changes in daily fruit and vegetable intake on risk of ischaemic heart disease, ischaemic stroke, and cancers of the colon, lung, stomach, and oesophagus [31]; changes in systolic blood pressure on risk of ischaemic heart disease and stroke [32]; and changes in BMI on risk of type 2 diabetes, ischaemic heart disease, hypertensive heart disease, ischaemic stroke, osteoarthritis, and cancers of the breast (in women), colon, lung, stomach, oesophagus, endometrium, kidney, and thyroid [33].

Since type 2 diabetes is itself a risk factor for ischaemic heart disease and stroke, we explicitly modelled the contribution of changing prevalence of type 2 diabetes (due to changing BMI) to the incidence of ischaemic heart disease and stroke, using relative risks of these diseases due to diabetes from the Asia Pacific Cohort Study [34]. To prevent double-counting, we reduced the relative risks of ischaemic heart disease and stroke until the total fraction of ischaemic heart disease and stroke attributable to excess BMI was equal to the sum of the fraction contributed directly from BMI and the fraction contributed from BMI via diabetes.

In addition, we reduced the excess risk of ischaemic heart disease and stroke that is associated with decreasing BMI by 50% [35], to account for mediation via systolic blood pressure.

### Population Modelling

We modelled the effect of changing incidence of diseases on the future health of the population using proportional multi-state lifetable modelling methods that we previously developed to evaluate the cost-effectiveness of preventive interventions in the Australian health care context [36–38]. Using these methods, we simulated the 2010 Australian population (by 5-y age and sex cohorts) over time until everyone was dead or had reached age 100 y. At each year, we determined incidence, prevalence, and mortality for each dietary-related disease, as well as mortality from all other causes. All model input data are presented in S1 Text.

From the changing incidence in disease, we determined the change in years of life lived by the Australian population due to each tax/subsidy intervention, and adjusted for time spent in ill health using disability weights from the Australian Burden of Disease Study [39], to determine the total disability-adjusted life years (DALYs) averted.

### Calculating Cost Implications

We determined the change in costs of treating disease over the lifetime of the Australian population by applying unit costs (e.g., cost per prevalent case of stroke) that we estimated, by age and sex, from Australian Institute of Health and Welfare data on disease costs and impacts [40]. We included changes in the costs of treating non-dietary-related diseases in the added years of life, but reported on these separately in the cost-effectiveness analysis.

The costs of implementing new taxation/subsidy interventions for foods in Australia was estimated to be $22 million (95% confidence interval: $19 million to $24 million) in 2010 Australian dollars, based on a previous estimate of costs for implementing changes to taxation in Australia [41]. This estimate included costs of basic administration, promotion through the media, and enforcement of the new tax/subsidy interventions. We assumed that these costs would be the same for all combinations of food taxes and subsidies and modelled them as a one-off up-front cost.

Since we were interested in evaluating cost-effectiveness from a health sector perspective, we did not include any costs to food manufacturers or retailers for product reformulation or labelling changes. We did calculate changes in household expenditure due to changes in product prices and consumption patterns with each tax/subsidy option (or combination); however, since these are a transfer payment, we report the changes in household expenditure separately and do not include them in the cost-effectiveness calculations.

### Cost-Effectiveness Analysis

We evaluated the cost-effectiveness of all 31 combinations of the five taxation and subsidy options. We simulated total DALYs and costs over the lifetime of the 2010 population, discounting future DALYs and costs back to the 2010 baseline year at a rate of 3%, in line with previous analyses of preventive interventions in Australia [36–38].

Each scenario was run 2,000 times in Monte Carlo analysis, to determine 95% uncertainty intervals (UIs) and probabilities of cost-effectiveness. The uncertainty in model input parameters (food consumption, proportion of purchased food that is wasted, food price elasticities, relative risks of disease, etc.) is presented in S2 Table. We present the probability of each tax and subsidy option being “dominant” (i.e., leading to an increase in health and net cost-savings) and being cost-effective against a threshold of AU$50,000 per DALY averted [36–38].

To determine the optimal package of tax and subsidy options, we calculated net monetary benefit [42] for all tax and subsidy combinations at cost-effectiveness thresholds between AU$0/DALY and AU$1 million/DALY. The optimal combination of the five tax and subsidy options was then determined by selecting intervention options in order of highest probability of maximum net benefit when incrementally added to the package at the AU$50,000 per DALY threshold.

### Scenario Analyses

We explored a number of possible demand- and supply-side responses to the imposition of food taxes and subsidies in scenario analyses. These included feasibility constraints on changes in total energy intake and total weight of foods consumed, food industry reformulation of foods to avoid taxes, and under- or over-shifting of price changes on taxed products.

For the constraint on energy intake, we restricted the change in average daily energy to ±250 kJ/d, which is approximately 3% of average daily energy intake, and roughly equivalent to the energy provided by a small apple. We did this by proportionally scaling food intake until the change in total daily energy did not exceed the target amount. Similarly, for the constraint on weight of foods consumed, we restricted the change in total mass of the diet to ±50 g/d, which is approximately 3% of the average weight of the current diet, and roughly equivalent to the weight of half of a medium-sized banana.

For the reformulation scenario, we estimated the impact if product manufacturers reduced the salt content of foods to the maximum recommended level [16], replaced oils high in saturated fat (e.g., palm oil) with oils lower in saturated fat (e.g., canola), replaced sugars in sugar-sweetened beverages with artificial sweeteners, and reduced sugars to no more than 10 g per 100 g in non-fruit-based products high in sugar (by reducing added sugars or replacing sugars with artificial sweeteners).

For the under-shifting of price changes, we assumed that price changes due to taxes would be reduced by 20% on products from the three companies with largest retail volume in Australia [38], assuming that these companies may have the greatest capacity to absorb costs. The proportion of all products from the three largest companies varies by food and drink category, ranging from 0% market share for around 60% of products up to more than 40% market share for sugar-sweetened beverages and some confectionery (S3 Table). We assumed that all other companies passed on the full price change due to taxes to the consumers. Our assumptions were similar for the over-shifting of price changes, but we assumed that price changes due to taxes were instead increased by 20%.

## Results

### Effect of Individual Tax and Subsidy Options on Population Risks

The effects of the tax and subsidy options on daily intake of fruits and vegetables, sodium, and total energy are shown in Table 2. Only the sugar tax led to improvements in all dietary measures—a reduction in sodium and energy intake and an increase in fruit and vegetable intake. The taxes on saturated fat, salt, and sugar-sweetened beverages all led to improvements in sodium and energy intake, but due to cross-price elasticity effects, there was an accompanying decrease in fruit and vegetable intake. The subsidy on fruits and vegetables led to an increase in fruit and vegetable intake, but an undesirable increase in sodium and energy intake.

**Table 2:** Change in dietary intake and diet cost with the individual tax and subsidy interventions.

Change in outcome variable	Saturated fat tax	Excess salt tax	Sugar-sweetened beverage tax	Fruit and vegetable subsidy	Sugar tax
Fruit and vegetable intake (g/day)	-5.4 (-8.4 to -2.8)	-3.7 (-4.7 to -2.8)	-3.1 (-4.5 to -1.8)	42.0 (37.9 to 46.0)	2.3 (0.6 to 3.9)
Sodium intake (mg/day)	-59 (-75 to -45)	-67 (-73 to -62)	-4 (-12 to 4)	48 (37 to 57)	-50 (-57 to -43)
Total energy intake (kJ/day)	-136 (-189 to -83)	-161 (-177 to -147)	-30 (-60 to -2)	236 (203 to 268)	-278 (-302 to -257)
Food and drink consumption (g/day)	-24 (-29 to -19)	-22 (-24 to -20)	-4 (-6 to -1)	50 (45 to 55)	-17 (-20 to -14)
Diet cost (%)	0.8% (0.1% to 1.5%)	-0.2% (-0.4% to -0.1%)	0.1% (-0.3% to 0.4%)	0.3% (-0.1% to 0.8%)	-0.6% (-0.9% to -0.3%)

NB. Values are mean and 95% uncertainty interval. Costs are presented in 2010 Australian dollars. The colours of the arrows indicate the direction of impact on disease for each dietary variable (green = favourable; red = unfavourable; orange = neutral).

### Cost-Effectiveness of Individual Tax and Subsidy Options

There was a big difference in outcomes between the subsidy intervention and the four tax interventions. When modelled over the lifetime of the population, the fruit and vegetable subsidy did not lead to an improvement in health or a reduction in disease treatment costs. The intervention was dominated, with only an 11% probability of being cost-effective against a AU$50,000 per DALY threshold.

In contrast to the subsidy intervention, the taxes on saturated fat, salt, sugar-sweetened beverages, and sugar all led to an improvement in population health, which ranged from 12,000 DALYs (95% UI: 2,100 to 21,000) averted for the sugar-sweetened beverage tax up to 270,000 DALYs averted (95% UI: 250,000 to 290,000) for the sugar tax (Table 3). Although the costs of treating dietary-related diseases were reduced with each of the taxes, this was partly countered by an increase in the costs of treating non-dietary-related diseases in the added years of life. Nevertheless, all of the tax interventions were cost-saving (dominant) for the health sector when all disease costs were included in the cost-effectiveness analysis.

**Table 3:** Lifetime population health impact, costs, and cost-effectiveness of the tax and subsidy interventions.

Outcome Variable	Saturated Fat Tax	Excess Salt Tax	Sugar-Sweetened Beverage Tax	Fruit and Vegetable Subsidy	Sugar Tax
**Health gain—DALYs averted**	97,000 (77,000 to 120,000)	130,000 (120,000 to 140,000)	12,000 (2,100 to 21,000)	−13,000 (−44,000 to 18,000)	270,000 (250,000 to 290,000)
**Costs (millions)**					
Intervention costs	$22 ($14 to $31)	$22 ($14 to $31)	$22 ($14 to $31)	$22 ($14 to $31)	$22 ($14 to $31)
Diet-related disease cost offsets	−$1,500 (−$2,100 to −$1,000)	−$2,000 (−$2,500 to −$1,500)	−$210 (−$390 to −$45)	$640 (−$160 to $1,500)	−$4,000 (−$5,000 to −$3,100)
Other disease costs*	$230 ($120 to $340)	$450 ($370 to $540)	−$70 (−$120 to −$20)	$1,500 ($1,200 to $1,900)	$1,300 ($1,200 to $1,500)
**Cost-effectiveness, including diet-related disease cost offsets**					
Median CER	Dominant	Dominant	Dominant	Dominated	Dominant
P(<$50,000/DALY)	100%	100%	99%	16%	100%
P(dominant)	100%	100%	98%	4%	100%
**Cost-effectiveness adding costs of other diseases**					
Median CER	Dominant	Dominant	Dominant	Dominated	Dominant
P(<$50,000/DALY)	100%	100%	99%	11%	100%
P(dominant)	100%	100%	99%	0%	100%

Values are presented as mean (95% uncertainty interval). Costs are presented in 2010 Australian dollars. A dominant intervention is an intervention that leads to a net health gain and net cost-savings.

*Costs of non-dietary-related diseases in added years of life. These included all conditions contributing to disease burden and health expenditure in the Australian population (e.g., mental, neurological, and musculoskeletal conditions) that were not directly related to changes in diet.

CER, cost-effectiveness ratio; DALY, disability-adjusted life year; P, probability.

### Cost-Effectiveness of Tax and Subsidy Combinations: The Optimal Package

**Fig 1:**
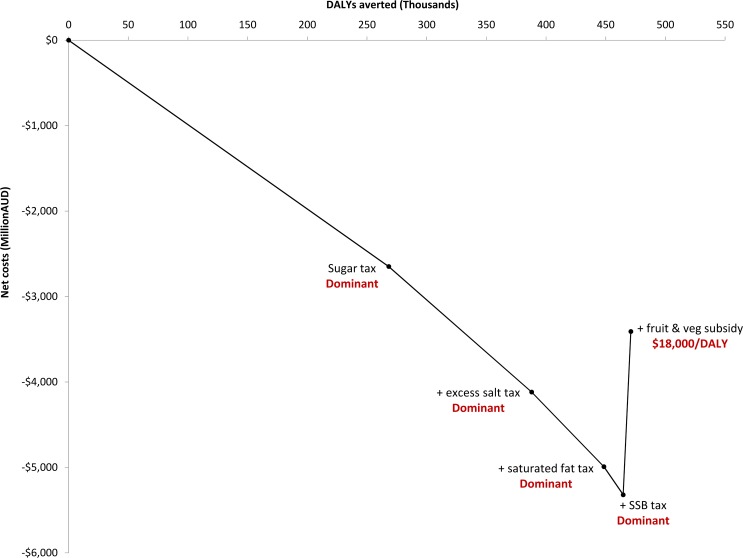
Cost-effectiveness of combining the tax and subsidy interventions. Each cost-effectiveness ratio reflects the cost-effectiveness of adding the intervention to the package. AUD, Australian dollars; DALY, disability-adjusted life year; SSB, sugar-sweetened beverage.

The highest net monetary benefit was achieved by implementing the sugar tax first, followed by the salt tax, saturated fat tax, sugar-sweetened beverage tax, and fruit and vegetable subsidy (Fig 1). Interestingly, the fruit and vegetable subsidy, although dominated when implemented on its own, was cost-effective when added to the combination of taxes. The median cost-effectiveness of adding the fruit and vegetable subsidy was AU$18,000/DALY, but the probability of being under the AU$50,000/DALY cost-effectiveness threshold was only 54% (S4 Table). The combination of all five tax and subsidy interventions led to an increase in population health of 470,000 DALYs (95% UI: 420,000 to 510,000) averted and a net cost-saving of AU$3.4 billion (AU$2.4 billion to AU$4.6 billion), and had a 100% probability of cost-savings.

### Scenario Analyses

The package of interventions was dominant under all scenarios we evaluated (Table 4). Constraining change in dietary energy intake to no more than 250 kJ/d (approximately 3% of average daily intake) reduced the health impact by 28%, although constraining change in the weight of foods consumed had no impact, because the changes were within the maximum of 50 g/d (approximately 3% of average daily consumption). A 20% over- or under-shifting of prices by the three largest product suppliers had only a ±3% impact on health outcomes. Food reformulation by product manufacturers—to reduce excess salt, replace sugars with artificial sweeteners, and replace unhealthy fats with healthier options—approximately doubled the potential population health gain.

**Table 4:** Change in the mean health impact and costs of the combined package of all tax and subsidy options under a range of scenarios.

Scenario	Health Gain (DALYs Averted)	Disease Cost Offsets (Millions)	Costs of Other Diseases (Millions)*	Cost-Effectiveness
**Combined intervention package (mean)**	470,000	−$6,800	$3,300	Dominant
**Diet energy constrained**	340,000 (−28%)	−$4,900 (−28%)	$2,400 (−28%)	Dominant
**Diet mass constrained**	470,000 (0%)	−$6,800 (0%)	$3,300 (0%)	Dominant
**Reformulation**	940,000 (+99%)	−$14,000 (+105%)	$6,900 (+105%)	Dominant
**Under-shift of price changes**	460,000 (−3%)	−$6,600 (−3%)	$3,300 (−2%)	Dominant
**Over-shift of price changes**	480,000 (+3%)	−$7,000 (+3%)	$3,400 (+2%)	Dominant

Values in parentheses reflect the percentage change in outcome for the scenario, relative to the mean outcome for the baseline scenario (shown in first row). Costs are presented in 2010 Australian dollars. A dominant intervention is an intervention that leads to net health gain and net cost-savings.

*Costs of non-dietary-related diseases in added years of life. These included all conditions contributing to disease burden and health expenditure in the Australian population (e.g., mental, neurological, and musculoskeletal conditions) that were not directly related to changes in diet.

DALY, disability-adjusted life year.

**Fig 2:**
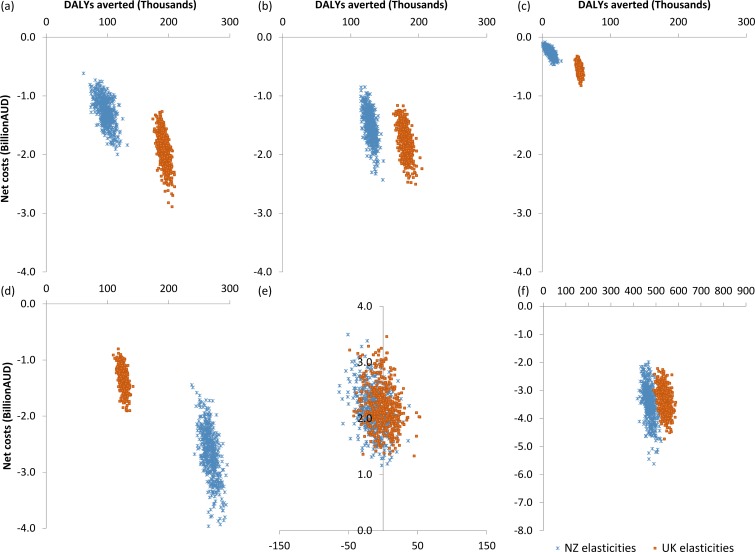
Comparison of cost-effectiveness with New Zealand and UK elasticity sets. Cost-effectiveness with New Zealand (blue) and UK (orange) elasticity sets for (A) saturated fat tax, (B) salt tax, (C) sugar-sweetened beverage tax, (D) sugar tax, (E) fruit and vegetable subsidy, and (F) package of all taxes/subsidies. AUD, Australian dollars; DALY, disability-adjusted life year; NZ, New Zealand.

When analyses were run with UK rather than New Zealand elasticities, all of the tax scenarios were still dominant and the subsidy intervention was still dominated (Fig 2). The health gain was around 1.5- to 5-fold greater for the saturated fat, salt, and sugar-sweetened beverage taxes, but around half the size for the sugar tax; the health gain for the fruit and vegetable subsidy was virtually identical. However, although there was variation in the magnitude of health gain with the two different sets of price elasticities, the effects largely cancelled each other out, such that, overall, there was relatively little difference when the tax and subsidy interventions were combined as a package (Fig 2F).

## Discussion

Our modelling using price elasticity data and a proportional multi-state lifetable model to simulate the health of the population into the future suggested that a combination of taxes on saturated fat, salt, sugar, and sugar-sweetened beverages and a subsidy on fruits and vegetables would very likely be cost-saving if implemented in Australia. The benefits to population health could be substantial. If implementing only one intervention, we found that any of the taxes could be a good option, leading to cost-savings for the health sector. Of the options we evaluated, the sugar tax was most likely to bring the biggest gains in health, followed by the salt tax, the saturated fat tax, and the sugar-sweetened beverage tax. However, we would not recommend the fruit and vegetable subsidy as an intervention on its own since our results suggest that it may lead to increased costs and no net health benefit for the population.

It is important to keep in mind that these results have used price elasticities derived in New Zealand, rather than Australia. While there are many similarities between the countries, in terms of geographical location, culture, history, income, diet, and product choices, there are still some differences. For example, Australia is a much larger island, with greater extremes of climate and a more urbanised population. These factors may influence the storage and transport of food, retail practices, product availability, and food quality, all of which may affect people’s preferences and ultimately their sensitivity to changes in prices. However, it is reassuring that when analysed with UK elasticities, where the dietary patterns and products are likely to be even more divergent, the cost-effectiveness of the tax and subsidy interventions and the combined health gain were similar, although there was some variation in health gain with individual taxes.

In addition to variations in diet and price sensitivity by country, the values of own- and cross-price elasticity will depend on the methods and assumptions applied in their derivation. For example, the elasticities derived in New Zealand were “conditional”, where it was assumed that there would be no change in the share of the household budget allocated for foods. While we did not assume there would be no change in food costs with the dietary interventions, we did set the magnitude of the interventions such that there would be less than 1% variation in total food expenditure for the average household. The largest impact of this was seen with the saturated fat tax, which led to a 0.8% increase in the cost of the diet (Table 2), which is equivalent to AU$0.15 per day (95% UI: AU$0.03 to AU$0.27).

While our modelling focused on the health implications of consumer responses to price changes, the imposition of taxes (or subsidies) also sends a signal to those in the food supply chain about a government’s commitment to tackling unhealthy diets [43]. We explored a number of possible supply-side responses, including variations in the pass-through of price changes and product reformulation. Reformulation to healthier products, so that they do not attract a tax (and therefore prices do not change for the consumer) is a good outcome from a health sector perspective. Our scenario reflects the maximum that might be achieved if manufacturers reformulate products that would be taxed (e.g., by reducing salt or replacing unhealthy fats and added sugars with healthier options). However, we do assume that the reformulation has no impact on consumer preferences (e.g., changes in taste or texture are not large enough to influence choices) and that food is not added to or manipulated in other ways that influence health (e.g., by reducing fibre). It is also possible that food manufacturers or retailers could use other strategies, such as marketing or price promotions to reduce or exaggerate the price effects, which we have not modelled.

Like our study, previous Australian analyses of a sugar-sweetened beverage tax [11] and a junk food tax [10] (similar to our combination of saturated fat, salt, sugar, and sugar-sweetened beverage taxes) indicated that the taxes could be cost-saving for the health sector. However, there were differences in the magnitude of the health gains. The sugar-sweetened beverage tax modelled by Veerman et al. [11] was approximately twice the size of our sugar-sweetened beverage tax. Taking this difference in the size of the tax into account, the effects on BMI were comparable in the two analyses; however, the DALY health gain was more than twice the size. Similarly, the health gain associated with the junk food tax modelled by Sacks et al. [10] was around 20% larger than the health gain from our comparable tax analyses (taking population size differences into account), although the effects on BMI were again comparable between the two analyses. There are a number of possible reasons for the differences between the studies. Both our current study and the previous studies used a proportional multi-state lifetable approach to model health outcomes, simulated outcomes over the lifetime of the Australian population, and presented results with a discount rate of 3%. However, there were important differences in the inclusion of cross-price elasticity effects and in the risk factor–disease relationships that were included in the health model. Veerman et al. measured cross-price elasticity effects on artificially sweetened beverages and modelled the health impacts of the changes in BMI that would stem from the beverage effects on energy intake. Sacks et al. measured cross-price elasticity effects on a broader range of foods, but only those that were considered to be unhealthy (e.g., biscuits, cakes, pastries, pies, snack foods, confectionery, and soft drinks), and, using an earlier version of the Veerman et al. model, they also modelled health impacts based on BMI changes stemming from energy intake effects. However, in this study, we measured cross-price elasticity effects across all foods and drinks in the diet (excluding infant foods and alcoholic beverages) and modelled the health impacts of changes in sodium intake and fruit and vegetable intake, in addition to changes in BMI stemming from energy intake effects. In our analyses of changes across the whole diet, the sugar-sweetened beverage tax led to a non-significant effect on sodium; however, it did lead to a reduction in fruit and vegetable intake, which countered the benefits from reduced BMI, lessening the health gain overall, in comparison to the previous studies.

The probability of harm associated with the fruit and vegetable subsidy (when implemented without accompanying unhealthy food taxes) in our modelling may at first appear counter-intuitive. Fruits and vegetables are generally high in fibre and have a low energy density, so if people substitute fruits and vegetables for relatively unhealthier foods, then measures of total fibre and energy intake will improve. However, using price subsidies or discounts as an incentive to purchase more fruits and vegetables may have the effect of increasing real income available to buy food, including unhealthy products, and could therefore lead to an overall increase in dietary measures such as saturated fat, sodium, or total energy intake. There is limited experimental evidence on the effects of price subsidies [44] to help us understand these potentially competing effects. In a review of experimental evidence on subsidies to promote healthy food purchases, An [45] identified only two studies (one in a university cafeteria and one in a high school cafeteria) that examined the effect of price discounts on fruits and/or vegetables. While both of these studies found an increase in the purchase of discounted fruit and vegetable items, neither study fully evaluated the effect on intake of undiscounted foods (e.g., unhealthy foods) to determine an overall impact on diet or energy intake.

The own- and cross-price elasticities reflect the change in the purchase of the products targeted by the subsidy (fruits and vegetables) and the average change in the purchase of all other foods in the diet. Our modelling using elasticities from New Zealand and the UK suggests that although fruit and vegetable purchases do increase in response to the price reduction, there is an accompanying increase in the purchase of other foods that are on average higher in energy and sodium. When we modelled the effects of these changes in energy, sodium, and fruit and vegetable intake on risks of diseases, and simulated the combined impact of all diseases on population health, we found that there was no net improvement in health (i.e., the benefits of increased fruit and vegetable intake were “cancelled out” by the harms from the increased energy and sodium intake). However, there are other factors that we did not include in this modelling, such as the health effects from changes in fibre, whole grains, and red and processed meats, which may alter the balance between benefit and harm.

There is still a great deal of uncertainty about the many possible causal pathways between diet and disease. The relative risks that quantify the dose–response relationships in our modelling are taken from meta-analyses of prospective cohort studies. While trial evidence may be preferred in establishing causality, trials also have drawbacks, notably non-adherence combined with misreporting. While the observational studies we have based our analyses on do adjust for potential confounding (e.g., age, sex, smoking), there is still potentially residual confounding from missing or poorly measured explanatory variables, although this may to some extent be offset by the countervailing regression dilution bias. Dietary measurement is far from perfect; random error (misclassification) in exposure measurements would theoretically lead to an underestimation of the true strength of an association.

The cross-price elasticities that so strongly influence how people spend their money when fruits and vegetables are subsidised will be influenced by a range of factors, such as habit, marketing, and cultural food preferences, and these factors will vary from one country to another. Interestingly, although we found differences in the types of foods and drinks that changed in response to a fruit and vegetable subsidy when using elasticities from two different countries (New Zealand and the UK), the overall effect on dietary parameters (e.g., saturated fat, energy, and sodium intake) was similar, and this led to similar cost-effectiveness outcomes. However, we really need more measurement studies of price elasticities in other countries that comprehensively and more uniformly address all categories of foods and drinks, before patterns can truly be discerned.

While the fruit and vegetable subsidy was cost-effective when added to the package of taxes in Australia, this may not be the case in other countries, where the typical diet and price elasticities may be different (e.g., in Asia) and where the costs of health care treatment may be different. In countries that might be expected to have similar per capita health benefits to those in Australia (e.g., in Western Europe and North America), the cost-effectiveness of adding the fruit and vegetable subsidy is likely to be more favourable in countries with higher health expenditure (and hence larger potential cost-savings from reducing disease) than in countries with lower health expenditure, and vice versa. The modelling methods used in this study could potentially be used in other countries to tailor a package of taxes and subsidies with the best probability of improving population health at the least cost to the government.

The package of taxes and subsidies we evaluated led to an average change in the price of food and drink products of 10%, which is only half the 20% that is often proposed as the minimum needed to have significant effects on obesity and cardiovascular disease [4]. Nevertheless, the outcome of 470,000 DALYs that we found could potentially be averted with the tax and subsidy package is greater than we have modelled for a wide range of other dietary interventions in Australia, including mandatory and voluntary regulation of salt in processed foods (breads, margarines, and cereals) [37], traffic light food labelling [10], community lifestyle programs [36], and individually tailored dietary counselling [38].

A citizens’ jury in Australia was in favour of using revenue from taxes to subsidise healthy food options [46]. It also gave strong support for taxation of sugar-sweetened beverages, but had reservations about applying taxes to processed meats and snack foods, concerned by the potential complexities of distinguishing between healthy and unhealthy options. Although there are concerns that food and drink taxes may unfairly impact poorer people (who spend proportionally more of their income on food and drink), economic modelling from Ireland [47] suggests that while a tax on unhealthy food may be regressive, when combined with a healthy food subsidy (as we have modelled here), the effect is poverty neutral.

While food taxes and subsidies are not currently on the political agenda in Australia, there is recurrent interest in broadening the goods and services tax, which would lead to price changes on a wide range of food and drink products [48], and this could be an excellent opportunity to introduce an accompanying package of taxes and subsidies to encourage healthier eating in Australia.

The food industry is unlikely to welcome policies that steer consumption away from profitable processed foods. Large companies or corporations can use their financial power to promote information that distracts and confuses the public, as well as influence research and lobby politicians [49]. Although such pressures did lead to a repeal of Denmark’s saturated fat tax, the continued implementation of taxes in other countries (e.g., Hungary, France, and Mexico) does indicate a growing political and public motivation to implement health-based food and drink taxes despite industry pressures. While food and beverage industries may seek to dismiss modelling studies such as this and rely only on the results of real-world population trials of tax or subsidy interventions, such studies are not currently available and take many years to complete. By synthesising the best available evidence, modelling studies can give an indication of whether it is worthwhile implementing and evaluating a change in policy around the pricing of foods and beverages, and can provide guidance on the design of the new policy and accompanying monitoring and evaluation strategies.

With large multi-national corporations playing a powerful advisory role in the negotiation of international trade and investment agreements, governments may be less willing to implement public health policies and programs that compel action from food manufacturers [50]. Given the huge global burden of dietary-related disease, and the rapid escalation that is occurring in low- and middle-income countries [1], it could be helpful if there was a legally binding global convention around diet, similar to the World Health Organization’s Framework Convention on Tobacco Control, to support and protect government rights to implement taxes and regulatory measures that will improve public health [50].

### Conclusion

This study adds to the growing evidence of large health benefits and cost-effectiveness of using taxes and regulatory measures to influence the consumption of healthy foods [51]. We believe that with such large potential health benefits for the Australian population, and large benefits in reducing health sector spending on the treatment of non-communicable diseases, the formulation of a tax and subsidy package should be given a more prominent role in Australia’s public health nutrition strategy.

## Supporting Information

S1 CHEERS Checklist CHEERS checklist. (PDF)

S1 Table The change in price of some typical products. (PDF)

S2 Table Model input parameter uncertainty. (PDF)

S3 Table Market share of the three largest product retailers (by retail volume), by food and drink category. (PDF)

S4 Table Cost-effectiveness of adding each intervention to a combined package, in order of cost-effectiveness. (PDF)

S1 Text Model input data. (PDF)

## Abbreviations

BMI: body mass index
DALY: disability-adjusted life year
UI: uncertainty interval
