# Supplementary material for: Taxes and Subsidies for Improving Diet and Population Health in Australia: A Cost-Effectiveness Modelling Study
Source: PLoS Med. 2017 Feb 14;14(2):e1002232. doi: 10.1371/journal.pmed.1002232 (PMC5308803; doi:10.1371/journal.pmed.1002232)
Supplement: S1 Table — (PDF) [file pmed.1002232.s002.pdf]

Table S1 The change in price of some typical products.

Supermarket product	Old price	New price	Change in price for a typical serve
Cadbury milk chocolate bar 50g	$2.02	$2.22	$0.20 per 50g bar
Nestle Peters Ice Cream Original Vanilla 2L	$6.09	$10.05	$0.20 per 100mL serve
Coca Cola Soft Drink Coke Bottle Chilled 600mL	$3.00	$3.28	$0.18 per 375mL can
Coles Sausages Classic Beef 560g	$3.30	$3.87	$0.19 per 2 thick sausages (188g)
McCain Pizza Family Supreme Frozen 500g	$6.53	$7.12	$0.19 per 2 slices of 30cm pizza (158g)
Coles Fresh Apples - Pink Lady Prepacked 1kg	$5.78	$4.37	-$0.20 per 1 medium apple (140g)

NB. All prices are shown in 2010 Australian dollars. The average change in price across all products was 10%.
