# Supplementary material for: Taxes and Subsidies for Improving Diet and Population Health in Australia: A Cost-Effectiveness Modelling Study
Source: PLoS Med. 2017 Feb 14;14(2):e1002232. doi: 10.1371/journal.pmed.1002232 (PMC5308803; doi:10.1371/journal.pmed.1002232)
Supplement: S2 Table — (PDF) [file pmed.1002232.s003.pdf]

Parameter	Distribution	Sources and assumptions
Relative risks (RRs) of disease	Normal (ln RR)	Relative risks due to change in blood pressure [1], type 2 diabetes [2], fruit and vegetable consumption [3] and body mass [4, 5].
Change in systolic blood pressure (mmHg) for each 100mmol/24h change in sodium intake	Normal	Parameters, by age, from models derived by Law et al [6].
Proportion of cardiovascular diseases mediated by systolic blood pressure	Beta	Parameter derived by Danaei et al [7]
Total intervention cost	Gamma	Cost includes basic administration, media and enforcement [8]. Standard error assumed to be 20% of point-estimate.
Disease cost offsets	Uniform	Cost per case derived from Australian treatment cost data [9] and rates of disease [10]. Range assumed to be ±25% of point-estimate.
Proportion of purchased food that is wasted	Beta	Proportions for 41 categories of foods and drinks [11, 12]
Food consumption	Normal	Consumption in g/day for 111 food and drink categories from Australian Health Survey [13]
Food price elasticities	Normal	Food price elasticity and cross-price elasticity values from Ni Mhurchu et al. [14]

NB. All costs adjusted to 2010 Australian dollars using Australian health price deflators [15], consumer price index [16] and/or purchasing power parities [17] where relevant.

References

1. Lewington S, Clarke R, Qizilbash N, Peto R, Collins R. Age-specific relevance of usual blood pressure to vascular mortality: a meta-analysis of individual data for one million adults in 61 prospective studies. Lancet. 2002;360(9349):1903-13. PubMed PMID: ISI:000179870200007.
2. Woodward M, Zhang X, Barzi F, Pan W, Ueshima H, Rodgers A, et al. The effects of diabetes on the risks of major cardiovascular diseases and death in the Asia-Pacific region. Diabetes Care. 2003;26(2):360-6.
3. Lock K, Pomerleau J, Causer L, McKee M. Low fruit and vegetable consumption. In: Ezzati M, Lopez A, Rodgers A, Murray C, editors. Comparative Quantification of Health Risks: Global and Regional Burden of Disease Attributable to Selected Major Risk Factors. Geneva: World Health Organisation; 2004.
4. James W, Jackson-Leach R, Mhurchu C, Kalamara E, Shayeghi M, Rigby N, et al. Overweight and obesity (high body mass index). In: Ezzati M, Lopez A, Rodgers A, Murray C, editors. Comparative Quantification of Health Risks: Global and Regional Burden of Disease Attributable to Selected Major Risk Factors. Geneva: World Health Organisation; 2004.
5. Renehan AG, Tyson M, Egger M, Heller RF, Zwahlen M. Body-mass index and incidence of cancer: a systematic review and meta-analysis of prospective observational studies. Lancet. 2008;371(9612):569-78. PubMed PMID: ISI:000253202600023.
6. Law MR, Frost CD, Wald NJ. By how much does dietary salt reduction lower blood-pressure? 1. Analysis of observational data among populations. Br Med J. 1991;302(6780):811-5. PubMed PMID: ISI:A1991FF37700020.

7. Danaei G, Ding EL, Mozaffarian D, Taylor B, Rehm J, Murray CJL, et al. The Preventable Causes of Death in the United States: Comparative Risk Assessment of Dietary, Lifestyle, and Metabolic Risk Factors. PLoS Med. 2009;6(4). doi: e1000058. PubMed PMID: ISI:000266214800010.

8. Cobiac LJ, Vos T, Doran C, Wallace A. Cost-effectiveness of interventions to prevent alcohol-related disease and injury in Australia. Addiction. 2009;104(10):1646-55.

9. AIHW. Disease costs and impacts study data. Canberra: Australian Institute of Health and Welfare, 2001.

10. Begg S, Vos T, Goss J, Barker B, Stevenson C, Stanley L, et al. The burden of disease and injury in Australia 2003. Canberra: Australian Institute of Health and Welfare, 2007.

11. Quested T, Ingle R, Parry A. Household Food and Drink Waste in the United Kingdom 2012. United Kingdom: WRAP, 2013.

12. Quested T, Murphy L. Household food and drink waste: A product focus. United Kingdom: WRAP, 2014.

13. ABS. Australian Health Survey Canberra: Australian Bureau of Statistics; 2011 [cited 2011 9 August]. Available from: http://www.abs.gov.au/websitedbs/D3310114.nsf/home/australian+health+survey.

14. Ni Mhurchu C, Eyles H, Schilling C, Yang Q, Kaye-Blake W, Genç M, et al. Food prices and consumer demand: differences across income levels and ethnic groups. PLoS ONE. 2013;8(10):e75934.

15. AIHW. Health expenditure Australia. Health & Welfare Expenditure Series. Canberra: Australian Institute of Health and Welfare, 2006.

16. ABS. Consumer Price Index, Australia, June quarter. Cat No. 6401.0. Canberra: Australian Bureau of Statistics, 2006.

17. OECD. Purchasing Power Parities (PPPs) for OECD Countries 1980-20062006 12 February 2006 [cited 2006 Feb 12]; (February 2006). Available from: www.oecd.org/std/ppp/.
