# Supplementary material for: Taxes and Subsidies for Improving Diet and Population Health in Australia: A Cost-Effectiveness Modelling Study
Source: PLoS Med. 2017 Feb 14;14(2):e1002232. doi: 10.1371/journal.pmed.1002232 (PMC5308803; doi:10.1371/journal.pmed.1002232)
Supplement: S3 Table — (PDF) [file pmed.1002232.s004.pdf]

Table S2 Market share of the three largest product retailers (by retail volume), by food and drink category

Category	Market share
Tea	0%
Coffee	0%
Juice	20%
Beverage flavourings sugar	10%
Beverage flavourings diet	0%
Drinks sugar	43%
Drinks diet	0%
Energy drinks sugar	23%
Energy drinks diet	0%
Beverage powder	0%
Rice and other cereal grains and starches - refined	0%
Rice and other cereal grains and starches - whole	0%
Regular breads, and bread rolls (plain/unfilled/untopped varieties) - refined	0%
Regular breads, and bread rolls (plain/unfilled/untopped varieties) - whole	0%
English-style muffins, flat breads, and savoury and sweet breads	0%
Pasta and pasta products	3%
Breakfast cereals, ready to eat	4%
Breakfast cereals, hot porridge style	0%
Sweet biscuits	6%
Savoury biscuits and crackers	25%
Cakes, buns, muffins, scones, cake-type desserts	8%
Sweet pastry products	0%
Pies, pasties and sausage rolls	0%
Savoury pastries other	0%
Breadcrumbs	0%
Batter products (pancakes, waffles, doughnuts, crumpets, coating, etc.)	0%
Butter and dairy blends	6%
Margarine	0%
Oils	4%
Fish and seafood fresh	0%
Fish and seafood canned/bottled	0%
Fish and seafood frozen and/or fried	8%
Pome fruit	0%
Berries	0%
Citrus fruit	0%
Stone fruit	0%
Tropical fruit	0%
Vine fruit	0%
Mixtures of two or more fruits	0%
Dried fruit, preserved fruit	0%
Fruit-based desserts	11%
Eggs	0%
Egg products and dishes	0%
Beef meat	0%
Lamb meat	0%

Pork meat	0%
Veal meat	0%
Game meat	0%
Poultry meat	0%
Liver, kidneys, tripe	0%
Sausages and frankfurts	0%
Bacon and deli meat	17%
Whole milk	29%
Reduced fat/skim milk	26%
Milk powder skim	17%
Yoghurt	18%
Cream	27%
Cheese	31%
Ice cream	31%
Dairy-based desserts	28%
Flavoured milk	35%
Milk substitute	0%
Milk substitute flavoured	0%
Milk substitute ice cream	25%
Meat alternatives	14%
Soup	7%
Seeds	0%
Nuts, nut butters, coconut milk, satay sauce	13%
Gravies and sauces	6%
Casserole base, powder	14%
Chutneys and relishes	0%
Dressings	12%
Stuffing	0%
Dips	17%
Potatoes plain	0%
Potatoes fried	0%
Cabbage, cauliflower and similar brassicas	0%
Carrot and similar root vegetables	0%
Leaf and stalk vegetables	0%
Peas and beans	0%
Tomatoes	0%
Other fruiting vegetables	0%
Mixed vegetables, onion and garlic	0%
Legumes/pulses dried	0%
Legume/pulse products	0%
Legumes/pulses canned	0%
Savoury snacks	0%
Sugar, honey and syrups	8%
Sweet spreads	0%
Sugar-based desserts	0%
Chocolate and chocolate-based confectionery	25%
Confectionery-type bars	9%
Cereal, fruit, nut and seed bars	21%
Other confectionery	44%

Meal replacement powders	0%
Savoury spreads and pastes	33%
Artificial sweetener	0%
Dried herbs and spices	0%
Salt	0%
Stock powder	17%
Stock liquid	0%
Baking powder	0%

NB. Market share determined from Euromonitor data for Australia
