# Supplementary material for: Taxes and Subsidies for Improving Diet and Population Health in Australia: A Cost-Effectiveness Modelling Study
Source: PLoS Med. 2017 Feb 14;14(2):e1002232. doi: 10.1371/journal.pmed.1002232 (PMC5308803; doi:10.1371/journal.pmed.1002232)
Supplement: S4 Table — (PDF) [file pmed.1002232.s005.pdf]

**Table S3** Cost-effectiveness of adding each intervention to a combined package, in order of cost-effectiveness

| | Sugar tax | + Salt tax | + Saturated fat tax | + SSB tax | + Fruit and veg subsidy |
|---|---|---|---|---|---|
| **Health gain** | | | | | |
| DALYs averted | 270,000 (250,000 to 290,000) | 120,000 (86,000 to 150,000) | 61,000 (12,000 to 110,000) | 16,000 (-39,000 to 74,000) | 6,400 (-46,000 to 61,000) |
| **Costs (millions)** | | | | | |
| Intervention costs | $22 ($14 to $31) | - | - | - | - |
| Diet-related disease cost offsets | -$4,000 (-$5,000 to -$3,100) | -$1,900 (-$3,700 to -$300) | -$940 (-$3,100 to $1,300) | -$280 (-$2,700 to $2,200) | $350 (-$1,800 to $2,700) |
| Other disease costs* | $1,300 ($1,200 to $1,500) | $410 ($150 to $690) | $67 (-$290 to $420) | -$45 (-$440 to $360) | $1,600 ($1,100 to $2,000) |
| **Cost-effectiveness** | | | | | |
| **Including diet-related disease cost offsets** | | | | | |
| Median CER | Dominant | Dominant | Dominant | Dominant | $4,000 |
| P(<$50,000/DALY) | 100% | 100% | 98% | 69% | 56% |
| P(Dominant) | 100% | 99% | 25% | 30% | 32% |
| **+ costs of other diseases** | | | | | |
| Median CER | Dominant | Dominant | Dominant | Dominant | $18,000 |
| P(<$50,000/DALY) | 100% | 100% | 98% | 69% | 54% |
| P(Dominant) | 100% | 97% | 0% | 2% | 3% |

NB. Values are mean and 95% uncertainty interval. Costs are presented in 2010 Australian dollars. SSB – sugar-sweetened beverage; DALY – disability-adjusted life year; CER – cost-effectiveness ratio; P – probability; Dominant – intervention leads to net health gain and net cost savings.
* Costs of other non-dietary-related diseases in added years of life. These included all conditions contributing to disease burden and health expenditure in the Australian population (e.g. mental, neurological, musculoskeletal, etc. conditions) that were not directly related to changes in diet.
