# Supplementary material for: Taxes and Subsidies for Improving Diet and Population Health in Australia: A Cost-Effectiveness Modelling Study
Source: PLoS Med. 2017 Feb 14;14(2):e1002232. doi: 10.1371/journal.pmed.1002232 (PMC5308803; doi:10.1371/journal.pmed.1002232)
Supplement: S1 Text — (PDF) [file pmed.1002232.s006.pdf]

		Mortality	Population	pYLD rate	Costs of other diseases per person
Male	0-4	0.00022	749611	0.017851	2424.803
	5-9	0.0001	701203	0.025258	1705.6
	10-14	0.0001	719763	0.030851	1416.849
	15-19	0.00048	770614	0.037447	1592.53
	20-24	0.00063	849732	0.04701	1759.573
	25-29	0.00073	844968	0.054421	1720.836
	30-34	0.00089	768096	0.059552	1679.462
	35-39	0.00111	802527	0.06453	1753.992
	40-44	0.00155	772142	0.072413	1918.6
	45-49	0.00239	781143	0.085938	2158.915
	50-54	0.0037	726885	0.097241	2491.038
	55-59	0.00544	655993	0.114223	3050.206
	60-64	0.0083	604200	0.142755	4112.211
	65-69	0.01353	450385	0.179923	5503.669
	70-74	0.02269	343844	0.223976	6493.424
	75-79	0.03859	256110	0.275803	7824.542
	80-84	0.06869	188982	0.334184	12125.93
	85-89	0.11839	99033	0.387401	16216.81
	90-94	0.19624	31669	0.417589	16216.81
	95-100	0.27033	7354	0.424698	16216.81
Female	0-4	0.00016	711477	0.013543	2024.091
	5-9	0.00008	664544	0.018105	1698.965
	10-14	0.00008	684015	0.026138	1687.305
	15-19	0.00022	730396	0.037577	2043.31
	20-24	0.00025	799927	0.049202	2486.21
	25-29	0.00032	820295	0.058481	2783.981
	30-34	0.00041	765947	0.062732	2802.529
	35-39	0.0006	813524	0.065327	2622.646
	40-44	0.00093	780524	0.071819	2589.954
	45-49	0.00146	793910	0.081276	2823.783
	50-54	0.00219	743491	0.092493	3133.079
	55-59	0.0032	670020	0.107171	3581.625
	60-64	0.00491	608337	0.118681	4574.696
	65-69	0.00794	459127	0.135817	5875.79
	70-74	0.01354	366600	0.172737	6443.799
	75-79	0.02353	295511	0.227012	7520.519
	80-84	0.04605	250324	0.287772	13260.74
	85-89	0.08873	168115	0.347742	19080.23
	90-94	0.16308	69159	0.386198	19080.23
	95-100	0.25208	22901	0.396103	19080.23

		Ischaemic heart disease				Ischaemic stroke			
		Incidence	Case Fatality	pYLD rate	$/prev	Incidence	Case Fatality	pYLD rate	$/prev
Male	0-4	-5.3E-23	0	0.03848	3574.206	0.0001	0.003698	0.328182	2688.053
	5-9	0.000001	0	0.03848	3574.206	0.000101	0.002714	0.328141	2688.053
	10-14	0.00001	0	0.03848	3574.206	0.000108	0.002366	0.328105	2688.053
	15-19	0.000021	0	0.03848	3574.206	0.000124	0.003041	0.328076	2688.053
	20-24	0.000019	0	0.03848	3574.206	0.000144	0.003707	0.328085	2688.053
	25-29	7.3E-05	0	0.03848	3574.206	0.000162	0.003927	0.328077	2688.053
	30-34	0.000261	8.67E-19	0.03848	3574.206	0.000191	0.004333	0.328078	2688.053
	35-39	0.000312	0.001166	0.03848	3574.206	0.000292	0.00476	0.328112	2688.053
	40-44	0.001708	0.011101	0.03848	3574.206	0.000398	0.005431	0.328177	2688.053
	45-49	0.004019	0.017067	0.03848	3574.206	0.000607	0.006934	0.328149	2688.053
	50-54	0.006341	0.014254	0.03848	3339.137	0.000971	0.008876	0.328211	3342.932
	55-59	0.009292	0.012538	0.03848	2751.463	0.001301	0.010857	0.328167	4980.131
	60-64	0.012643	0.01303	0.03848	2320.696	0.001729	0.014826	0.328202	7069.32
	65-69	0.016414	0.014646	0.03848	2125.291	0.00291	0.021078	0.368876	9836.496
	70-74	0.0198	0.018138	0.03848	1971.368	0.00417	0.032226	0.368922	12299.85
	75-79	0.022425	0.024451	0.042066	1879.668	0.006443	0.051922	0.271511	14307.48
	80-84	0.024877	0.036271	0.049004	1796.185	0.010506	0.082745	0.27164	16343.26
	85-89	0.026957	0.061692	0.060203	1725.028	0.014921	0.136999	0.271916	18421.27
	90-94	0.028256	0.106839	0.076593	1682.334	0.019373	0.230059	0.272278	19668.07
	95-100	0.029215	0.163006	0.076593	1682.334	0.023729	0.308849	0.272446	19668.07
Female	0-4	-5.3E-23	0	0.041789	2209.941	0.0001	0.000558	0.201578	1401.228
	5-9	0.000001	0	0.041789	2209.941	0.000101	0.002616	0.201313	1401.228
	10-14	0.00001	0	0.041789	2209.941	0.000108	0.003744	0.201242	1401.228
	15-19	0.00002	0	0.041789	2209.941	0.000123	0.002866	0.201195	1401.228
	20-24	4.8E-05	0	0.041789	2209.941	0.000165	0.001781	0.201348	1401.228
	25-29	0.000111	0	0.041789	2209.941	0.000255	0.001758	0.201323	1401.228
	30-34	0.000156	0	0.041789	2209.941	0.00034	0.002017	0.201286	1401.228
	35-39	0.000355	0	0.041789	2209.941	0.000318	0.003426	0.201218	1401.228
	40-44	0.000754	0	0.041789	2209.941	0.000314	0.005139	0.201206	1401.228
	45-49	0.001343	1.3E-05	0.041789	2209.941	0.000534	0.006159	0.201248	1401.228
	50-54	0.002147	0.000612	0.041789	2134.773	0.000841	0.006226	0.201344	1625.392
	55-59	0.003735	0.001779	0.041789	1946.852	0.000916	0.007117	0.201291	2185.802
	60-64	0.007173	0.00289	0.041789	1852.113	0.001103	0.012364	0.201257	3249.805
	65-69	0.010009	0.00752	0.060412	1897.149	0.002018	0.019274	0.272339	5069.199
	70-74	0.013341	0.013106	0.064991	1916.739	0.003034	0.030303	0.272414	8098.683
	75-79	0.017864	0.020105	0.069253	1898.162	0.005523	0.050308	0.392565	12943.3
	80-84	0.022616	0.033445	0.074113	1912.665	0.01013	0.083418	0.392725	17428.75
	85-89	0.026153	0.062751	0.083424	1976.788	0.015054	0.15278	0.392953	21375.44
	90-94	0.027108	0.119302	0.104627	2015.262	0.020187	0.295432	0.393226	23743.46
	95-100	0.029835	0.203507	0.104627	2015.262	0.024588	0.50574	0.393271	23743.46

		Type 2 diabetes				Breast cancer			
		Incidence	Case Fatality	pYLD rate	$/prev	Incidence	Case Fatality	pYLD rate	$/incid
Male	0-4	0	0	0.072198	608.0495	-	-	-	-
	5-9	0	0	0.072198	608.0495	-	-	-	-
	10-14	0.000005	0	0.072198	608.0495	-	-	-	-
	15-19	0.000017	0.000414	0.072198	608.0495	-	-	-	-
	20-24	0.00004	0.000856	0.072198	608.0495	-	-	-	-
	25-29	0.000263	0.000384	0.072198	608.0495	-	-	-	-
	30-34	0.001277	0.000367	0.072198	608.0495	-	-	-	-
	35-39	0.00296	0.000525	0.072198	608.0495	-	-	-	-
	40-44	0.005285	0.000484	0.072198	608.0495	-	-	-	-
	45-49	0.00798	0.00036	0.072198	608.0495	-	-	-	-
	50-54	0.010271	0.000506	0.075494	645.7944	-	-	-	-
	55-59	0.011585	0.001018	0.077559	740.1568	-	-	-	-
	60-64	0.012695	0.001622	0.081478	821.442	-	-	-	-
	65-69	0.01377	0.002535	0.084103	883.1114	-	-	-	-
	70-74	0.01523	0.003741	0.084674	890.2894	-	-	-	-
	75-79	0.019288	0.004796	0.085179	815.7302	-	-	-	-
	80-84	0.027189	0.005931	0.085285	760.0253	-	-	-	-
	85-89	0.035444	0.007818	0.085544	732.6018	-	-	-	-
	90-94	0.036235	0.009135	0.084632	716.1477	-	-	-	-
	95-100	0.03201	0.009876	0.082643	716.1477	-	-	-	-
Female	0-4	0.000001	0	0.07255	610.3057	0	0	0.123629	14989.98
	5-9	0	0.000024	0.07255	610.3057	0	0	0.123629	14989.98
	10-14	0	0	0.07255	610.3057	0	0.0002	0.123629	14989.98
	15-19	0.00001	0.00185	0.07255	610.3057	0	0.0013	0.123629	14989.98
	20-24	0.000105	0.003423	0.07255	610.3057	5.42E-20	0.0022	0.123629	14989.98
	25-29	0.000507	0.0008	0.07255	610.3057	0.0001	0.0115	0.123629	14989.98
	30-34	0.001389	0.000209	0.07255	610.3057	0.0002	0.0355	0.123629	14989.98
	35-39	0.002245	0.00025	0.07255	610.3057	0.0005	0.0387	0.123629	14989.98
	40-44	0.002972	0.000263	0.07255	610.3057	0.001	0.0306	0.123629	14989.98
	45-49	0.003702	0.000419	0.07255	610.3057	0.0016	0.0239	0.123629	14989.98
	50-54	0.004615	0.000533	0.074898	671.4057	0.0023	0.0198	0.123629	14524.2
	55-59	0.005913	0.000684	0.076339	824.1557	0.0029	0.0177	0.123629	13359.74
	60-64	0.007523	0.001159	0.078134	935.0976	0.0033	0.016	0.123629	12930.23
	65-69	0.009003	0.001922	0.080012	983.3274	0.0034	0.0139	0.123629	13603.16
	70-74	0.010722	0.002822	0.080803	989.4985	0.0033	0.0126	0.123629	14271.62
	75-79	0.014076	0.003781	0.080336	932.5816	0.0032	0.0131	0.123629	14933.38
	80-84	0.020047	0.004991	0.079539	822.1623	0.0031	0.0151	0.123629	15285.58
	85-89	0.026108	0.006643	0.07862	631.4895	0.0031	0.0185	0.123629	15173.43
	90-94	0.026003	0.008263	0.079373	517.0858	0.0029	0.0226	0.123629	15106.14
	95-100	0.019895	0.009072	0.079311	517.0858	0.0026	0.0255	0.123629	15106.14

		Colorectal cancer				Lung cancer			
		Incidence	Case Fatality	pYLD rate	$/incid	Incidence	Case Fatality	pYLD rate	$/incid
Male	0-4	0	0	0.122493	21102.79	0	0.0085	0.100804	20729.44
	5-9	0	0	0.122493	21102.79	0	0.006	0.100804	20729.44
	10-14	0	0	0.122493	21102.79	0.0001	0.0022	0.100804	20729.44
	15-19	0	0	0.122493	21102.79	0.0002	0.0006	0.100804	20729.44
	20-24	0	0	0.122493	21102.79	0.0002	0.001	0.100804	20729.44
	25-29	0	0	0.122493	21102.79	0.0001	0.0012	0.100804	20729.44
	30-34	0	0	0.122493	21102.79	0.0001	0.0014	0.100804	20729.44
	35-39	0.0001	0.003	0.122493	21102.79	0.0001	0.0041	0.100804	20729.44
	40-44	0.0001	0.0248	0.122493	21102.79	0.0001	0.014	0.100804	20729.44
	45-49	0.0003	0.0511	0.122493	21102.79	1E-04	0.0378	0.100804	20729.44
	50-54	0.0006	0.0616	0.122493	21143.03	0.0004	0.0785	0.100804	20146.57
	55-59	0.0012	0.0595	0.122493	21243.64	0.0009	0.1219	0.100804	18689.37
	60-64	0.0019	0.0534	0.122493	21426.42	0.0016	0.152	0.100804	18099.27
	65-69	0.0029	0.049	0.122493	21732.48	0.0025	0.185	0.100804	18809.82
	70-74	0.0039	0.0452	0.122493	21885.46	0.0034	0.2173	0.100804	18965.81
	75-79	0.0048	0.0417	0.122493	21808.85	0.0042	0.2366	0.100804	18289.97
	80-84	0.0054	0.0392	0.122493	22064.82	0.0047	0.2464	0.100804	17367.92
	85-89	0.0059	0.0383	0.122493	22819.69	0.0049	0.2533	0.100804	16076.57
	90-94	0.0062	0.04	0.122493	23272.6	0.0051	0.2556	0.100804	15301.76
	95-100	0.0064	0.0399	0.122493	23272.6	0.0052	0.2536	0.100804	15301.76
Female	0-4	0	0	0.113144	20676.09	0	0.0001	0.108051	21728.66
	5-9	0	0	0.113144	20676.09	0	0.0001	0.108051	21728.66
	10-14	0	0	0.113144	20676.09	0	0.0002	0.108051	21728.66
	15-19	0	0	0.113144	20676.09	0	0.0028	0.108051	21728.66
	20-24	0	0	0.113144	20676.09	0	0.0063	0.108051	21728.66
	25-29	0	0	0.113144	20676.09	0	0.0078	0.108051	21728.66
	30-34	0	0	0.113144	20676.09	0	0.0137	0.108051	21728.66
	35-39	0	0.0076	0.113144	20676.09	0	0.0301	0.108051	21728.66
	40-44	0.0001	0.038	0.113144	20676.09	1E-04	0.088	0.108051	21728.66
	45-49	0.0003	0.058	0.113144	20676.09	0.0002	0.14	0.108051	21728.66
	50-54	0.0005	0.0542	0.113144	20486.19	0.0003	0.1457	0.108051	21327.28
	55-59	0.0008	0.0484	0.113144	20011.44	0.0005	0.156	0.108051	20323.84
	60-64	0.0012	0.0459	0.113144	19941.09	0.0008	0.191	0.108051	20400.67
	65-69	0.0018	0.0434	0.113144	20477.36	0.0011	0.2049	0.108051	22097.91
	70-74	0.0025	0.0405	0.113144	20828.48	0.0014	0.2131	0.108051	22695.66
	75-79	0.0032	0.0396	0.113144	20901.85	0.0016	0.224	0.108051	21644.18
	80-84	0.0038	0.0412	0.113144	20749.26	0.0018	0.2266	0.108051	19382.1
	85-89	0.0043	0.0437	0.113144	20257.71	0.0018	0.2227	0.108051	15304.13
	90-94	0.0046	0.0448	0.113144	19962.78	0.0019	0.2155	0.108051	12857.35
	95-100	0.0047	0.0443	0.113144	19962.78	0.0019	0.207	0.108051	12857.35

		Gastric cancer				Oesophagus cancer			
		Incidence	Case Fatality	pYLD rate	$/incid	Incidence	Case Fatality	pYLD rate	$/incid
Male	0-4	0	0	0.061237	26868.17	0	0	0.589841	34916.33
	5-9	0	0	0.061237	26868.17	0	0	0.589841	34916.33
	10-14	0	0	0.061237	26868.17	0	0	0.589841	34916.33
	15-19	0	0	0.061237	26868.17	0	0	0.589841	34916.33
	20-24	0	0.0001	0.061237	26868.17	0	0	0.589841	34916.33
	25-29	0	0.0007	0.061237	26868.17	0	0	0.589841	34916.33
	30-34	0	0.0027	0.061237	26868.17	0	0	0.589841	34916.33
	35-39	0	0.0065	0.061237	26868.17	0	0.0348	0.589841	34916.33
	40-44	0	0.0133	0.061237	26868.17	0	0.1104	0.589841	34916.33
	45-49	0.0001	0.02	0.061237	26868.17	0.0001	0.1938	0.589841	34916.33
	50-54	1E-04	0.0266	0.061237	26560.16	0.0001	0.2641	0.589841	34968.98
	55-59	0.0002	0.0381	0.061237	25790.11	1E-04	0.3727	0.589841	35100.62
	60-64	0.0003	0.0537	0.061237	25959.72	0.0002	0.5071	0.589841	38096.49
	65-69	0.0004	0.0715	0.061237	27538.8	0.0003	0.6923	0.589841	45388.69
	70-74	0.0007	0.0826	0.061237	27538.84	0.0004	1.0069	0.589841	46596.96
	75-79	0.0008	0.0876	0.061237	25170.31	0.0005	1.4644	0.589841	38679.33
	80-84	0.001	0.0942	0.061237	23481.42	0.0006	1.8839	0.589841	31748.46
	85-89	0.0012	0.1032	0.061237	22811.99	0.0006	1.9914	0.589841	26297.74
	90-94	0.0014	0.1127	0.061237	22410.34	0.0007	2	0.589841	23027.31
	95-100	0.0016	0.1148	0.061237	22410.34	0.0007	2	0.589841	23027.31
Female	0-4	0	-2.7E-20	0.060295	32913.39	0	0	0.21578	32289.06
	5-9	0	0.0004	0.060295	32913.39	0	0	0.21578	32289.06
	10-14	0	0.0004	0.060295	32913.39	0	0	0.21578	32289.06
	15-19	0	0.0004	0.060295	32913.39	0	0	0.21578	32289.06
	20-24	0	0.0004	0.060295	32913.39	0	0	0.21578	32289.06
	25-29	0	0.0004	0.060295	32913.39	0	0	0.21578	32289.06
	30-34	0	0.0026	0.060295	32913.39	0	0	0.21578	32289.06
	35-39	0	0.0102	0.060295	32913.39	0	0	0.21578	32289.06
	40-44	0	0.0152	0.060295	32913.39	0	0.0003	0.21578	32289.06
	45-49	0	0.0182	0.060295	32913.39	0	0.0086	0.21578	32289.06
	50-54	0.0001	0.0218	0.060295	31838.57	0	0.0398	0.21578	36364.33
	55-59	0.0001	0.0276	0.060295	29151.52	0.0001	0.0826	0.21578	46552.52
	60-64	1E-04	0.0472	0.060295	27269.21	0.0001	0.0978	0.21578	48117.3
	65-69	0.0002	0.0561	0.060295	26594	1E-04	0.1229	0.21578	36746.96
	70-74	0.0003	0.0661	0.060295	25933.54	0.0002	0.1731	0.21578	32136.22
	75-79	0.0004	0.0824	0.060295	25295.17	0.0002	0.2495	0.21578	37664.88
	80-84	0.0005	0.1	0.060295	23514.21	0.0003	0.3486	0.21578	36326.96
	85-89	0.0006	0.1231	0.060295	20019.35	0.0004	0.4643	0.21578	24689.17
	90-94	0.0007	0.1415	0.060295	17922.43	0.0005	0.6211	0.21578	17706.49
	95-100	0.0008	0.1332	0.060295	17922.43	0.0005	0.864	0.21578	17706.49

		Endometrial cancer				Kidney cancer			
		Incidence	Case Fatality	pYLD rate	$/incid	Incidence	Case Fatality	pYLD rate	$/incid
Male	0-4	-	-	-	-	0	0.0002	0.040292	19664.75
	5-9	-	-	-	-	0	0.0006	0.040292	19664.75
	10-14	-	-	-	-	0	0.0005	0.040292	19664.75
	15-19	-	-	-	-	0	0.0002	0.040292	19664.75
	20-24	-	-	-	-	0	0.0002	0.040292	19664.75
	25-29	-	-	-	-	0	0.0008	0.040292	19664.75
	30-34	-	-	-	-	0	0.0042	0.040292	19664.75
	35-39	-	-	-	-	0	0.0142	0.040292	19664.75
	40-44	-	-	-	-	0.0001	0.0228	0.040292	19664.75
	45-49	-	-	-	-	1E-04	0.0271	0.040292	19664.75
	50-54	-	-	-	-	0.0002	0.0305	0.040292	19774.11
	55-59	-	-	-	-	0.0003	0.0349	0.040292	20047.5
	60-64	-	-	-	-	0.0004	0.0402	0.040292	19728.15
	65-69	-	-	-	-	0.0006	0.0421	0.040292	18519.68
	70-74	-	-	-	-	0.0008	0.0407	0.040292	17741.09
	75-79	-	-	-	-	0.0009	0.0384	0.040292	17607.32
	80-84	-	-	-	-	0.001	0.0365	0.040292	15800.62
	85-89	-	-	-	-	0.0011	0.0369	0.040292	11484.53
	90-94	-	-	-	-	0.0011	0.0414	0.040292	8894.877
	95-100	-	-	-	-	0.0011	0.0514	0.040292	8894.877
Female	0-4	0	0	0.031734	12867.41	0	0	0.047292	18707.54
	5-9	0	0	0.031734	12867.41	0	0	0.047292	18707.54
	10-14	0	0	0.031734	12867.41	0	0.0001	0.047292	18707.54
	15-19	0	0	0.031734	12867.41	0	0.0009	0.047292	18707.54
	20-24	0	0	0.031734	12867.41	0	0.0053	0.047292	18707.54
	25-29	0	0	0.031734	12867.41	0	0.0121	0.047292	18707.54
	30-34	0	1E-04	0.031734	12867.41	0	0.0139	0.047292	18707.54
	35-39	0	0.0017	0.031734	12867.41	0	0.0164	0.047292	18707.54
	40-44	0.0001	0.0094	0.031734	12867.41	0	0.0161	0.047292	18707.54
	45-49	1E-04	0.0157	0.031734	12867.41	0.0001	0.0143	0.047292	18707.54
	50-54	0.0002	0.0151	0.031734	12683.39	1E-04	0.0153	0.047292	18914.55
	55-59	0.0004	0.0132	0.031734	12223.34	0.0002	0.019	0.047292	19432.08
	60-64	0.0005	0.0121	0.031734	13037.25	0.0002	0.0245	0.047292	19928.48
	65-69	0.0005	0.0117	0.031734	15762.11	0.0003	0.0318	0.047292	20393.17
	70-74	0.0006	0.0118	0.031734	16450.67	0.0004	0.0372	0.047292	20687.79
	75-79	0.0006	0.0124	0.031734	14084.8	0.0005	0.0403	0.047292	20727.32
	80-84	0.0006	0.0136	0.031734	13366.38	0.0005	0.045	0.047292	19542.83
	85-89	0.0006	0.0153	0.031734	15119.14	0.0005	0.0515	0.047292	16522.31
	90-94	0.0007	0.0176	0.031734	16170.8	0.0006	0.0539	0.047292	14709.99
	95-100	0.0007	0.0204	0.031734	16170.8	0.0006	0.053	0.047292	14709.99

		Hypertensive heart disease				Thyroid cancer			
		Incidence	Case Fatality	pYLD rate	$/prev	Incidence	Case Fatality	pYLD rate	$/incid
Male	0-4	0	0	0.091839	15809.84	0	0.0001	0.022211	10397.54
	5-9	0	0	0.091839	15809.84	0	0.0003	0.022211	10397.54
	10-14	0	0	0.091839	15809.84	0	0.0003	0.022211	10397.54
	15-19	0	0.13	0.091839	15809.84	0	0.0002	0.022211	10397.54
	20-24	0	0.13	0.091839	15809.84	0	0.0002	0.022211	10397.54
	25-29	0	0.13	0.091839	15809.84	0	0.0001	0.022211	10397.54
	30-34	0	0.13	0.091839	15809.84	0	0.0001	0.022211	10397.54
	35-39	0	0.13	0.091839	15809.84	0	0.0001	0.022211	10397.54
	40-44	0	0.13	0.091839	15809.84	0	0.0001	0.022211	10397.54
	45-49	0	0.13	0.091839	15809.84	0	1E-04	0.022211	10397.54
	50-54	0	0.13	0.091839	18537.9	0.0001	0.0002	0.022211	11138.88
	55-59	0	0.13	0.091839	25358.04	0.0001	0.0014	0.022211	12992.25
	60-64	1E-04	0.13	0.091839	27191.37	0.0001	0.0058	0.022211	14645.12
	65-69	0.0002	0.13	0.091839	21544.5	0.0001	0.0087	0.022211	15997.24
	70-74	0.0003	0.13	0.091839	16495.96	0.0001	0.013	0.022211	16160.09
	75-79	0.0006	0.13	0.091839	12344.89	0.0001	0.0164	0.022211	14539
	80-84	0.0014	0.13	0.091839	8299.162	0.0001	0.0159	0.022211	14662.58
	85-89	0.0031	0.13	0.091839	4411.428	1E-04	0.0149	0.022211	17403.18
	90-94	0.0052	0.13	0.091839	2078.788	0.0002	0.0123	0.022211	19047.53
	95-100	0.0052	0.13	0.091839	2078.788	0.0002	0.0052	0.022211	19047.53
Female	0-4	0	0	0.072293	26660.85	0	0	0.017968	8835.281
	5-9	0	0	0.072293	26660.85	0	0	0.017968	8835.281
	10-14	0	0	0.072293	26660.85	0	0	0.017968	8835.281
	15-19	0	0	0.072293	26660.85	0	0	0.017968	8835.281
	20-24	0	0	0.072293	26660.85	5.42E-20	5.42E-20	0.017968	8835.281
	25-29	0	0.13	0.072293	26660.85	0.0001	0.0001	0.017968	8835.281
	30-34	0	0.13	0.072293	26660.85	0.0001	0	0.017968	8835.281
	35-39	0	0.13	0.072293	26660.85	0.0001	0	0.017968	8835.281
	40-44	0	0.13	0.072293	26660.85	1E-04	0	0.017968	8835.281
	45-49	0	0.13	0.072293	26660.85	0.0002	0.0002	0.017968	8835.281
	50-54	0	0.13	0.072293	29061.42	0.0002	0.0005	0.017968	9077.435
	55-59	0	0.13	0.072293	35062.85	0.0001	0.0007	0.017968	9682.82
	60-64	0.0001	0.13	0.072293	35843.38	0.0001	0.0014	0.017968	10976.98
	65-69	1E-04	0.13	0.072293	28792.6	0.0001	0.0019	0.017968	13304.29
	70-74	0.0002	0.13	0.072293	21972.04	0.0001	0.0024	0.017968	14324.86
	75-79	0.0006	0.13	0.072293	15496.84	0.0001	0.0035	0.017968	13385.33
	80-84	0.0015	0.13	0.072293	9698.493	0.0001	0.0058	0.017968	12221.77
	85-89	0.0036	0.13	0.072293	4915.444	0.0001	0.0075	0.017968	10722.18
	90-94	0.0069	0.13	0.072293	2045.615	0.0001	0.0038	0.017968	9822.422
	95-100	0.0103	0.13	0.072293	2045.615	0.0001	0.0011	0.017968	9822.422

		Osteoarthritis			
		Incidence	Case Fatality	pYLD rate	$/prev
Male	0-4	0	0	0.0748	5346.349
	5-9	0	0	0.0748	5346.349
	10-14	0	0	0.0748	5346.349
	15-19	0	0	0.0748	5346.349
	20-24	5.42E-20	0	0.0748	5346.349
	25-29	0.0001	0	0.0748	5346.349
	30-34	0.0003	0	0.0748	5346.349
	35-39	0.0005	0	0.0748	5346.349
	40-44	0.0009	0	0.0748	5346.349
	45-49	0.0013	0	0.0748	5346.349
	50-54	0.0019	0	0.0748	5346.349
	55-59	0.0029	0	0.0748	5346.349
	60-64	0.0046	0	0.0748	5346.349
	65-69	0.0063	0	0.060356	5346.349
	70-74	0.0085	0	0.056691	5346.349
	75-79	0.0099	0	0.054513	5346.349
	80-84	0.0098	0	0.050987	5346.349
	85-89	0.0093	0.0002	0.047085	5346.349
	90-94	0.0089	0.0005	0.045136	5346.349
	95-100	0.0088	0.0011	0.044961	5346.349
Female	0-4	0	0	0.082734	5346.349
	5-9	0	0	0.082734	5346.349
	10-14	0	0	0.082734	5346.349
	15-19	0	0	0.082734	5346.349
	20-24	0	0	0.082734	5346.349
	25-29	0	0	0.082734	5346.349
	30-34	0.0001	0	0.082734	5346.349
	35-39	1E-04	0	0.082734	5346.349
	40-44	0.0004	0	0.082734	5346.349
	45-49	0.001	0	0.082734	5346.349
	50-54	0.0018	0	0.082734	5346.349
	55-59	0.0033	0	0.082734	5346.349
	60-64	0.0058	0	0.082734	5346.349
	65-69	0.0085	0	0.067888	5346.349
	70-74	0.0119	0	0.061645	5346.349
	75-79	0.0145	0.0001	0.057887	5346.349
	80-84	0.0153	1E-04	0.05496	5346.349
	85-89	0.0151	0.0003	0.052959	5346.349
	90-94	0.0147	0.0008	0.052685	5346.349
	95-100	0.0146	0.0016	0.053196	5346.349
